# Development and validation of a generic methyltransferase enzymatic assay based on an SAH riboswitch

**DOI:** 10.1016/j.slasd.2024.100161

**Published:** 2024-05-22

**Authors:** Ha Pham, Meera Kumar, Anibal Ramos Martinez, Mahbbat Ali, Robert G. Lowery

**Affiliations:** a BellBrook Labs, Madison, WI, USA; b Thermo Fisher Scientific, Madison WI, USA

**Keywords:** Epigenetics, Histone methyltransferase, DNA methyltransferase, Methyltransferase assay, Aptamer, Riboswitch, RNA methyltransferase, HTS assay

## Abstract

Methylation of proteins and nucleic acids plays a fundamental role in epigenetic regulation, and discovery of methyltransferase (MT) inhibitors is an area of intense activity. Because of the diversity of MTs and their products, assay methods that detect *S*-adenosylhomocysteine (SAH) – the invariant product of *S*-adenosylme-thionine (SAM)-dependent methylation reactions - offer some advantages over methods that detect specific methylation events. However, direct, homogenous detection of SAH requires a reagent capable of discriminating between SAH and SAM, which differ by a single methyl group. Moreover, MTs are slow enzymes and many have submicromolar affinities for SAM; these properties translate to a need for detection of SAH at low nanomolar concentrations in the presence of excess SAM. To meet these needs, we leveraged the exquisite molecular recognition properties of a naturally occurring SAH-sensing RNA aptamer, or riboswitch. By splitting the riboswitch into two fragments, such that SAH binding induces assembly of a trimeric complex, we engineered sensors that transduce binding of SAH into positive fluorescence polarization (FP) and time resolved Förster resonance energy transfer (TR-FRET) signals. The split riboswitch configuration, called the AptaFluor^™^ SAH Methyltransferase Assay, allows robust detection of SAH (Z’ > 0.7) at concentrations below 10 nM, with over-night signal stability in the presence of typical MT assay components. The AptaFluor assay tolerates diverse MT substrates, including histones, nucleosomes, DNA and RNA, and we demonstrated its utility as a robust, enzymatic assay method for several methyltransferases with SAM K_m_ values < 1 μM. The assay was validated for HTS by performing a pilot screen of 1,280 compounds against the SARS-CoV-2 RNA capping enzyme, nsp14. By enabling direct, homogenous detection of SAH at low nanomolar concentrations, the AptaFluor assay provides a universal platform for screening and profiling MTs at physiologically relevant SAM concentrations.

## 1. Introduction

Methylation of histones, DNA, and RNA is an important epigenetic regulatory modification that has been implicated in diverse therapeutic areas including metabolic diseases, cardiovascular, cancer, inflammation/autoimmunity and neurodegeneration [1–5]. In humans, there are three DNA methyltransferases (DNMTs) that modify DNA at CpG sites, 57 known RNA methyltransferase (RNMTs) that methylate mRNAs, microRNAs, ribosomal RNAs, and tRNAs at all four bases, and at least 35 active histone methyltransferases (HMTs) that modify histones at specific Lys-or Arg-residues [6,7]. Histone and DNA methylation dynamically interact to regulate gene expression by controlling the accessibility of DNA to the transcriptional machinery. Methylation of mRNA controls its stability, translation, localization and splicing, with feedback on transcription and translation via methylation of microRNAs and ribosomal RNAs, respectively [3,8,9].

Along with the other epigenetic ‘writers’ and ‘erasers’ that control gene expression, methyltransferases have been the focus of intense drug discovery efforts over the past ten years [10]. There are currently three FDA-approved drugs targeting epigenetic MTs: two DNA methyltransferases (DNMTs) inhibitors, azacytidine and decitabine, originally developed as chemotherapeutics rather than epigenetic drugs; and one histone methyltransferase inhibitor, tazemetostat, which targets the lysine HMT, EZH2; all are used for various types of cancer [10]. There are dozens of ongoing clinical trials involving DNA and histone methyltransferase inhibitors, and the first RNMT inhibitor, for METTL3/METTL14, recently entered Phase I trials [11].

Methyltransferases present a number of challenges from an assay development standpoint. They are generally very slow enzymes, with turnovers of less than 0.1 min^−1^ in many cases, and they tend to have low K_m_ values for SAM, many in the sub-micromolar range [12]. These properties impose very high sensitivity requirements on enzymatic assay methods, i.e., detection of 5–20 nM of product under conditions typically used for screening (i.e., initial velocity, sub-saturating SAM). The high cost of enzymes is another driver for more sensitive detection methods, as some MTs function as complexes, with three or four proteins required for full activity.

Though there is great diversity amongst methyl acceptors, virtually all known mammalian methyltransferases use SAM as a methyl donor, and detection of SAH, the invariant product, is advantageous over detection of methylated products for a number of reasons. HMTs can add two methyl groups at arginines and as many as three at lysines, resulting in a total of six possible methylation states. The diversity of methylated reaction products combined with variability in surrounding amino acids complicates immunochemical assay methods, as a single antibody generally does not recognize all of the products formed by a single HMT [12]. Moreover, the development of specific antibodies is not keeping pace with the discovery of new methylation sites, and assay development is being prevented in some cases. Methyl binding domains typically have very low affinity, and thus do not afford sensitive detection. Radiometric or mass spectral detection of methylated peptides have the required sensitivity and have been used successfully for MT inhibitor profiling [13,14], but they require a significant investment in specialized equipment, facilities or and/or regulatory certification.

SAH detection provides a much simpler, universal MT assay method, however significant technical gaps have thus far prevented development of robust, highly sensitive SAH detection assays. We and others have developed antibodies capable of discriminating between SAH and SAM [15,16], but they lack the affinity and specificity required for robust detection of SAH at levels below 100 nM in the presence of excess SAM. Indirect SAH detection via enzyme-coupled assays has become the primary approach, i.e., enzymatic conversion of SAH to a molecule that can be detected directly by absorbance, fluorescence or luminescence [17–24]. A common enzyme-coupled assay strategy involves conversion of SAH to homocysteine and AMP by SAH hydrolase. Homocysteine can be detected by thiol-sensitive fluors, and AMP can be detected by competitive fluorescence polarization immunoassay or by luminescence, following further enzymatic steps to produce ATP [20–22,24]. However, none of these methods have the sensitivity required for detection of SAH in the low nanomolar range, and the use of multiple coupling enzymes, as many as four in some cases, increases the risk for interference from screening compounds.

To overcome the technical gap in MT HTS assays, we used a naturally occurring SAH-binding RNA aptamer [25,26], or “riboswitch” to develop a TR-FRET-based SAH sensor. Riboswitches are RNA-based sensors that bacteria use to transduce fluctuations in key metabolites into changes in the expression of related metabolic enzymes [27]. Riboswitches for nucleotides are not uncommon, but the affinity and specificity of the SAH riboswitches stands out, e.g. a K_d_ of 20 nM for SAH and an affinity for SAM that is at least 1000-fold lower [26]. This exquisite selectivity is at least partly explained by binding and structural analyses showing that virtually every functional group in the SAH molecule interacts with the SAH riboswitch [25]. Prior efforts to use an SAH riboswitch as the basis for an MT assay have not fulfilled the requirements for an HTS enzymatic assay, either because the biosensor was designed for in use in vivo [28] or because modification of the riboswitch decreased its affinity for SAH [29].

Our initial strategy for assay development was to leverage the native riboswitch signaling mechanism by transducing the conformational switch that occurs upon SAH binding into a fluorescent signal. We developed an FP-based assay using fluorescently-labelled reporter oligonucleotides that annealed to the riboswitch specifically when SAH was bound. Though this resulted in a functional assay, it did not meet our sensitivity and selectivity criteria for an HMT assay even after extensive optimization efforts.

Noting that a split aptamer approach had been used to increase the sensitivity of DNA aptamer-based biosensors for estradiol and ATP [30, 31], we tested variants of a split SAH riboswitch and identified a pair of aptamer fragments that bound SAH with affinity and selectivity similar to the intact riboswitch. The split SAH aptamer was used to develop homogenous MT enzyme assays using FP and TR-FRET readouts.

## 2. Materials and methods

### Reagents.

AptaFluor detection reagents were produced at BellBrook Labs; the Aptafluor^®^ SAH Methyltransferase TR-FRET assay reagents are available in kit format (Part # 3023), including 10x Enzyme Stop Reagent (6 % sodium-dodecyl sulfate), 10x SAH Detection Buffer (200 mM Tris (pH 8.5), 200 mM MgCl_2_, 3 M NaCl, 0.2 % Brij-35, 10 mM urea), P1-Biotin, P2-Dylight 650, Tb-Streptavidin, and Conjugation Buffer. P1-Biotin is a 55-base RNA oligomer with a biotin molecule at the 5′ end. It is incubated with Tb-Streptavidin to produce P1-Terbium, the donor piece of the split SAH aptamer.

#### Enzymes:

Recombinant human PRMT5-MEP50 Enzyme Complex was from Sigma Aldrich (Cat. #SRP0146); Recombinant human PRMT4 and DOT1L were from Reaction Biology (Cat. #HMT-11–120, #HMT-11–101); Recombinant human METTL3/METTL14 was from Active Motif, (Cat. #31499, #31570). Recombinant human nsp14 was from Abcam (Cat. #ab277616).

#### Methyl Acceptors:

Nucleosomes (HeLa Mono/Di) were from Reaction Biology (Cat. #HMT-35–123). Histone H2A, full length human, was from Sigma Aldrich (Cat. #SRP0406); Histone H3 peptide (aa 1–21) was from Anaspec (Cat. #AS-61701). G(5′)ppp(5′)A RNA Cap Structure Analog was from New England BioLabs Inc. (Cat. #S1406L). ssRNA Oligomer (5′-GUUGCCUGUUCGUGUUGGACUUGCCUGU-3′) was from IDT.

#### Inhibitors:

GSK591, GSK3235025, JNJ64619178 were from Sell-eckChem. Sinefungin was from Millipore Sigma. UZH2, STM2457 were from MedChemExpress. The bioactive library of 1280 compounds was from Tocris (Tocriscreen 2.0, part # 7151).

### Enzymatic Assays.

All enzymatic assays were performed in white Corning 384-well, round bottom, low volume, polystyrene, non-binding surface microplates (Cat. # 4513) in a 10 μl volume. Mixing after additions was performed by orbital shaking for 40 s. PRMT3 was incubated at 30 °C for 2 hr in 50 mM Tris (pH 8.5), 5 mM MgCl_2_ 100 mM NaCl, 500 nM SAM and 10 μM Histone H4 peptide (aa 1–20). DOT1L was incubated at 30 °C for 120 min in 50 mM Tris (pH 8.5), 5 mM MgCl_2_, 100 mM NaCl, 0.01 % Triton X-100, 110 nM SAM and 25 μg/mL (Hela Mono/Di) Nucleosomes. PRMT4 was incubated at 30 °C for 120 min in 20 mM Tris (pH 8.5), 5 mM MgCl_2_, 100 mM NaCl, 1 mM DTT, 245 nM SAM and 1 μM Histone H3 peptide. PRMT5 was incubated at 30 °C for 90 min in 20 mM Tris (pH 8), 2 mM MgCl_2_, 1 mM EDTA, 1 mM DTT, 0.01 % Triton X-100, 5 μM SAM and 5 μM Histone H2A. METTL3/METTL14 was incubated at 30 °C for 2 h in 20 mM Tris (pH 7.5), 0.5 mM MgCl_2_, 0.01 % BSA, 0.01 % Tween-20, 0.02 U/μL RNAseOUT, 1 mM DTT, 250 nM SAM, and 1 μM RNA substrate. Nsp14 was incubated at 37 °C for 90 min in 20 mM Tris (pH 8), 3 mM MgCl_2_, 0.01 % BSA, 1 mM EDTA, 1 mM DTT, 100 nM SAM, and 50 μM G(5′)ppp(5′)A. Enzyme titrations were performed on two separate days to determine the optimal concentration for a robust signal that is sensitive to inhibition while maintaining initial velocity conditions, i.e., a concentration that resulted in 70–80 % maximal response (EC_70_-EC_80_). Optimized enzyme concentrations were subsequently used for inhibitor dose response experiments (PRMT5, METTL3/METTL14, nsp14) and the pilot screen (nsp14).

Aptafluor^®^ SAH Methyltransferase TR-FRET Assays were performed as end point reactions in duplicate or triplicate in 10 μl volume. 5 μL of Enzyme Stop Mix containing 0.6 % sodium dodecylsulfate in SAH Detection Buffer (20 mM Tris (pH 8.5), 20 mM MgCl_2_, 300 mM NaCl, 0.02 % Brij-35, 1 mM urea) was added to quench enzyme reactions. Then, 5 μL of SAH Detection Mix containing 20 nM P1-Terbium, 40 nM P2-Dylight in SAH Detection Buffer was added to the reaction. Note that the P1-Terbium Mix was prepared by preincubating P1-Biotin and Tb-Streptavidin in Conjugation buffer at room temperature for 15 min before adding to the SAH Detection Mix. Finally, the assay was equilibrated at RT for 3 h before reading the plate. Plates were read on either a PHERAstar (BMG Labtech, Germany) equipped with a 337 nm excitation wavelength, dual emission at 620/665 nm, and a delay time of 50 μs, or a M-1000 plate reader (Tecan, Switzerland) featuring a 317 nm excitation wavelength, dual emission at 617/665 nm, and a delay time of 60 μs. Signal is expressed as Δ 665 nm/620 nm × 1000. Note that the absolute values of the TR-FRET signals were lower in the Tecan plate reader compared to the PHERAstar, but the Z’ values were similar.

Standard curves mimicking MT enzyme reactions were used to determine SAH concentrations from TR-FRET signals. Mock reactions mimicking enzymatic conversion (0, 1.0, 2.5, 5..0, 7.5, etc.……100 %) of SAM to SAH were dispensed into wells in 10 μL of the corresponding MT reaction buffer, followed by the addition of Enzyme Stop Mix and AptaFluor detection reagents as described above. For each standard curve, total [SAM + SAH] remained constant, representing the initial SAM concentrations used in the MT enzyme reactions. TR-FRET signals were measured after 3 hour at ambient temperature and Δ 665 nm/620 nm × 1000 was plotted vs SAH concentration using a four-parameter nonlinear regression curve fitting in GraphPad Prism, and the quantity of SAH produced was interpolated.

### Pilot Screen and Interference Screen.

1280 compounds comprising the Tocriscreen 2.0 collection of pharmacologically active compounds were dispensed in 384- well assay-ready plates at the UW-Madison Small Molecule Screening Facility using an Echo Acoustic Liquid Handler (Beckman-Coulter) at 10 μM assay concentration, *n* = 1. For the pilot screen, 1.45 nM nsp14 was incubated at 37 °C for 90 min in 20 mM Tris (pH 8.0), 3 mM MgCl_2_, 0.01 % BSA, 1 mM EDTA, 1 mM DTT, 100 nM SAM, and 50 μM RNA Cap Substrate. To allow identification of compounds that interfere with the AptaFluor detection reagents, compounds were screened under identical conditions, but nsp14 was omitted, and reactions contained 90 nM SAM and 10 nM SAH to mimic completed nsp14 reactions. For both screens, compounds that resulted in TR-FRET signals greater than three standard deviations (SDs) from the mean were considered hits. Assay robustness was assessed by determining the Z and Z’ factor, with reactions lacking enzyme as the negative control.

## 3. Results and discussion

### Assay Development.

**Fig. 1.**
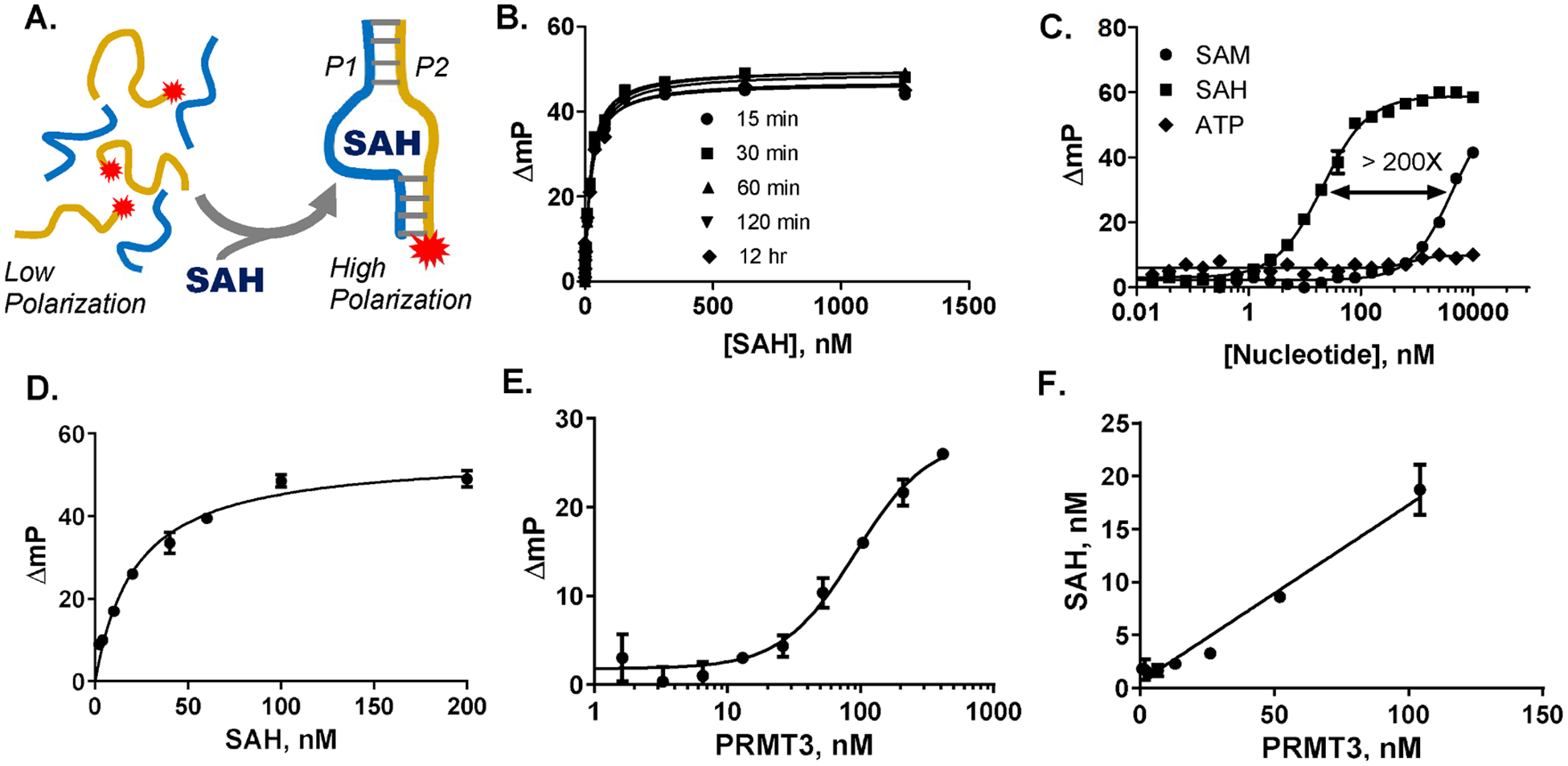
Split aptamer SAH assay with FP readout. A. SAH dependent assembly of split aptamer (Dar1 P1/P2) causes increase in polarization of a fluor attached to the P2 element. B. SAH titration, showing concentration dependence and stability of signal. C. Comparison of response to SAH, SAM and ATP. D. Standard curve mimicking enzymatic conversion of 200 nM SAM to SAH. E. Titration of PRMT3 in the presence of 500 nM SAM and Histone H4 peptide substrate. F. Conversion of FP data to SAH formation demonstrating linear dependence on enzyme concentration.

Based on a literature review and preliminary testing, an SAH riboswitch from *D. aromatic*a (Dar-1) was chosen for development of a split aptamer-based assay [26]. The predicted folding and imputed SAH binding interactions [26] suggested two different versions of a split Dar-1 riboswitch, both comprised of a longer 5′ part (P1) and the remaining 3′ element (P2). We tested these configurations with a far red fluor on the shorter P2 element, allowing FP-based detection (Fig. 1A). One of the split aptamer constructs enabled detection of SAH with a K_d_ of 22 nM and greater than 200-fold selectivity vs. SAM (Fig. 1B, C, D), and we validated it for enzyme detection using PRMT3 (Fig. 1E, F). Though the FP-based assay enabled detection of PRMT3 using 100 nM SAM, which was our functional milestone, the maximum FP shift observed was less than 30 mP (Fig. 1E), and, even at 200 nM SAM, Z’ values did not exceed 0.5 until 30 % of the SAM was converted to SAH (Fig. 1D). Generally, a shift of at least 100 mP @ ≤ 10 % substrate conversion is desirable for HTS. It is likely that the FP signal is limited by the relatively modest difference in size between the free P2 RNA and the liganded P1–P2 complex. In this regard, attachment of one half of a split DNA aptamer to a silver nanoparticle produced a lactoferrin sensor with a signal greater than 100 mP [32]; to our knowledge, this is the only other example of a split aptamer-based assay with an FP readout. Attempts to use a similar approach by attaching Streptavidin to the P1 oligomer were not successful (data not shown).

**Fig. 2.**
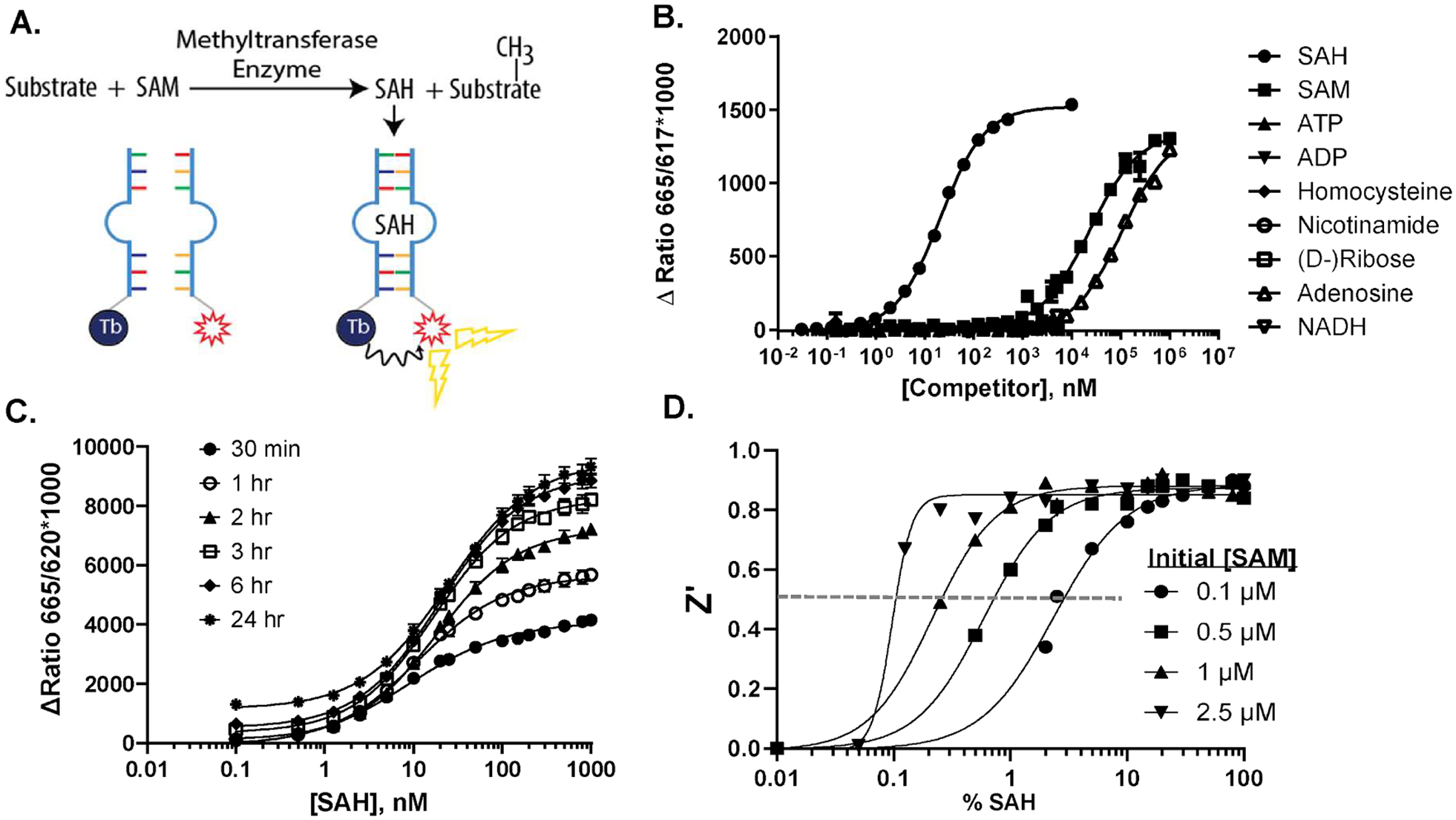
Split aptamer SAH assay with TR-FRET readout. A. AptaFluor^™^ SAH methyltransferase assay principle. SAH induces assembly of a split RNA aptamer, bringing FRET donor and acceptor together to produce a TR-FRET signal. B. Assay response and selectivity. Equilibrium binding assays with SAH, SAM and related nucleotides were performed (*n* = 3); plates were read at 3 hr. EC_50_ values for SAH, SAM, and adenosine were 0.02, 23, and 108 μM, respectively. C. Time to equilibrium and signal stability. An SAM/SAH standard curve starting at 1 μM SAM was set up (*n* = 12) and plates were read at the indicated times. D. Assay robustness. SAM/SAH standard curves starting at the indicated SAM concentrations were set up (*n* = 12) and Z’ values were calculated for each data point, relative to 0% SAH.

To configure the Dar-1 split aptamer for TR-FRET-based detection, Tb and Eu lanthanide chelates were attached to the P1 element as donors, and four different organic fluors with excitation spectra overlapping the lanthanide emission (Alexa 633, Alexa 647, Cy5, and Dylight 650) were attached to the P2 oligo as acceptors (Fig. 2A). The Tb/Dylight 650 combination produced the best signal:background and was used for further assay development. The sensitivity and selectivity of the TR-FRET-based assay were similar to the FP format, and the signal magnitude was sufficient for use in HTS (Fig. 2B); moreover, using a highly purified preparation of SAM increased the observed selectivity to more than 1000-fold. Monitoring a standard curve over time indicated that formation of the SAH-split aptamer complex requires more than three hours to reach equilibrium at room temperature, which precludes use of the assay for continuous enzyme detection (Fig. 2C). Though we did not take any special precautions to prevent RNA degradation other than using nuclease-free water for all buffers, the signal was stable for at least 24 hr., which will facilitate use of the split aptamer in automated HTS workflows. (Note that an RNase inhibitor was used in the METTL3/METTL14 assays (Fig. 5.A.–C.) to prevent degradation of the RNA substrate.) To test the capability for using the assay with low concentrations of SAM, we constructed standard curves mimicking enzymatic conversion of SAM to SAH, starting at initial SAM concentrations ranging from 100 nM to 2.5 μM, with sufficient replicates to calculate Z’ values 2D). Z’ values exceeded 0.5 at less than 1 % SAH for all but the 100 SAM curve, which exceeded the 0.5 threshold at less than 3 % SAH nM). These results clearly show that the TR-FRET based split aptamer assay, which we called AptaFluor, has the sensitivity required for screening MTs with SAM K_m_ values well below 1 μM.

### Enzyme Detection.

**Fig. 3.**
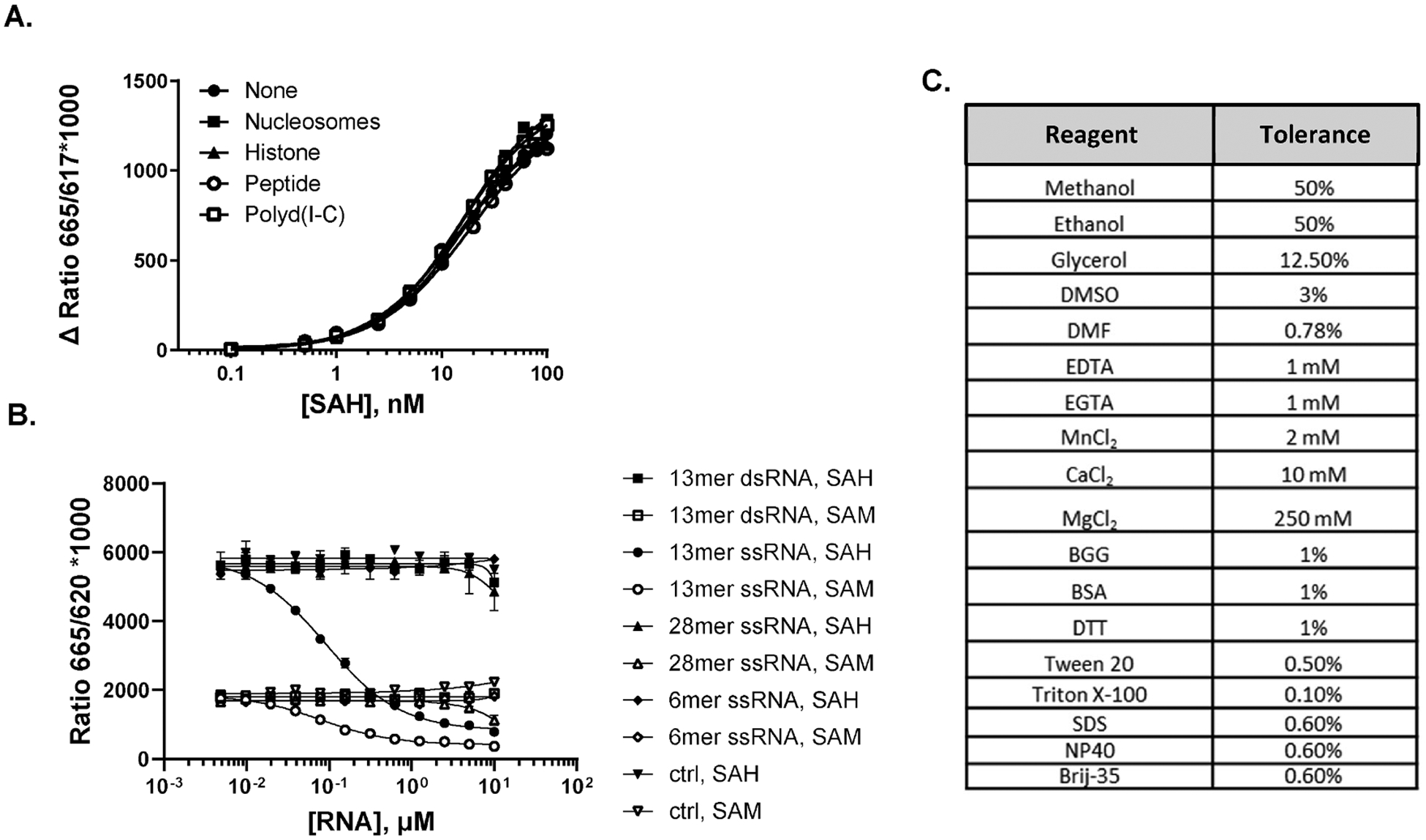
A. Tolerance of DNA and protein substrates. SAM/SAH standard curves starting at 100 nM SAM were set up in the presence of nucleosomes (10 ng/μL), histone H3.3 (3 ng/μL), histone H3 peptide [1–21] (10 μM), and polyd(I-C) (2.5 mU/μL); control wells lacked a MT substrate. B. Tolerance of RNA substrates. RNA titrations were performed in the presence of AptaFluor detection reagents and 100 nM SAH or 100 nM SAM; control wells lacked RNAs. C. Tolerance of various reagents. Tolerance is defined as the maximum concentration that resulted in < 10% change in the assay signal for 10 nM SAH.

Prior to testing enzymes, we screened MT substrates and commonly used reagents for interference with the AptaFluor SAH assay. HMTs have diverse substrate requirements in vitro; some function well with short peptides, while others require full length histones, or even intact nucleosome complexes; e.g., DOT1L and Nsd2 [23]. The AptaFluor Assay was unaffected by working concentrations of any of the HMT substrates tested, including nucleosomes, which are a heterogenous, partially purified cell fraction (Fig. 3A). DNMTs and RNMTs methylate both single and double strand nucleic acid substrates, and the potential for interference with the AptaFluor detection reagents by base pairing is obvious. Though not an exhaustive analysis, we found that most of the nucleic acids tested had little or no effect on the AptaFluor detection reagents at concentrations up to 10 μM (Fig. 3A, Though one ssRNA 13-mer interfered potently, presumably by disrupting assembly of the split aptamer-SAH complex, the 6-mer or 28-mer comprised of the same repeated sequence had little effect (Fig. 3B); therefore the interference is quite specific. We next tested reagents that are commonly used in enzyme reactions and/or HTS, including salts, chelators, detergents and organic solvents (Fig. 3C); the criteria for interference was ≥ 10 % decrease in signal for detection of 10 nM SAH. In general, reagents were very well tolerated, with the notable exception of the chelators EDTA and EGTA, which is not surprising as they are known to react with the Tb complex used as a TR-FRET donor. the toleration of SDS up to 0.6 % is beneficial as it will allow quenching of many enzyme reactions by denaturation. It is not clear why MgCl_2_ is tolerated so much better than MnCl_2_; a possible explanation is Mn-induced intrinsic RNA cleavage [33]. Though Mg^2+^ has been reported to increase the affinity of a closely related SAH aptamer several-fold [25], it caused only a two-fold increase in the affinity of the split Dar1 aptamer at concentrations of 25 mM or higher (data not shown).

To demonstrate the utility of the AptaFluor assay, we selected enzymes of therapeutic interest with diverse methyl acceptors and SAM K_m_ values ranging from less than 100 nM to low micromolar: three histone methyltransferases, DOT1L, PRMT4 and PRMT5 and two RNA methyltransferases, METTL3/METTL14 and nsp14. DOTL1 catalyzes methylation of lysine 79 of histone H3 and requires intact nucleosomes for optimal activity [23]. PRMT4 and PRMT5 catalyze methylation at arginine residues in peptide and native histone substrates, respectively [23]. For PRMT5, we used a WRAD complex with MEP50, which imparts high affinity substrate recognition and full catalytic activity [34]. METTL3/METTL14 methylates adenosine at the N^6^ position (m6A) in RNA substrates with a DRACH motif; METTL3 is the catalytic subunit and METTL14 facilitates substrate recognition [34]. nsp14 catalyzes the methylation of viral RNAs at the N7 position of guanosine in G(5′) ppp (5′)A substrates [35]. For all enzymes, we used SAM concentrations that were at or near the K_m_ values, determined internally or drawn from the literature (Table 1), and methyl acceptors at a concentration of at least 2 × K_m_.

**Fig. 4.**
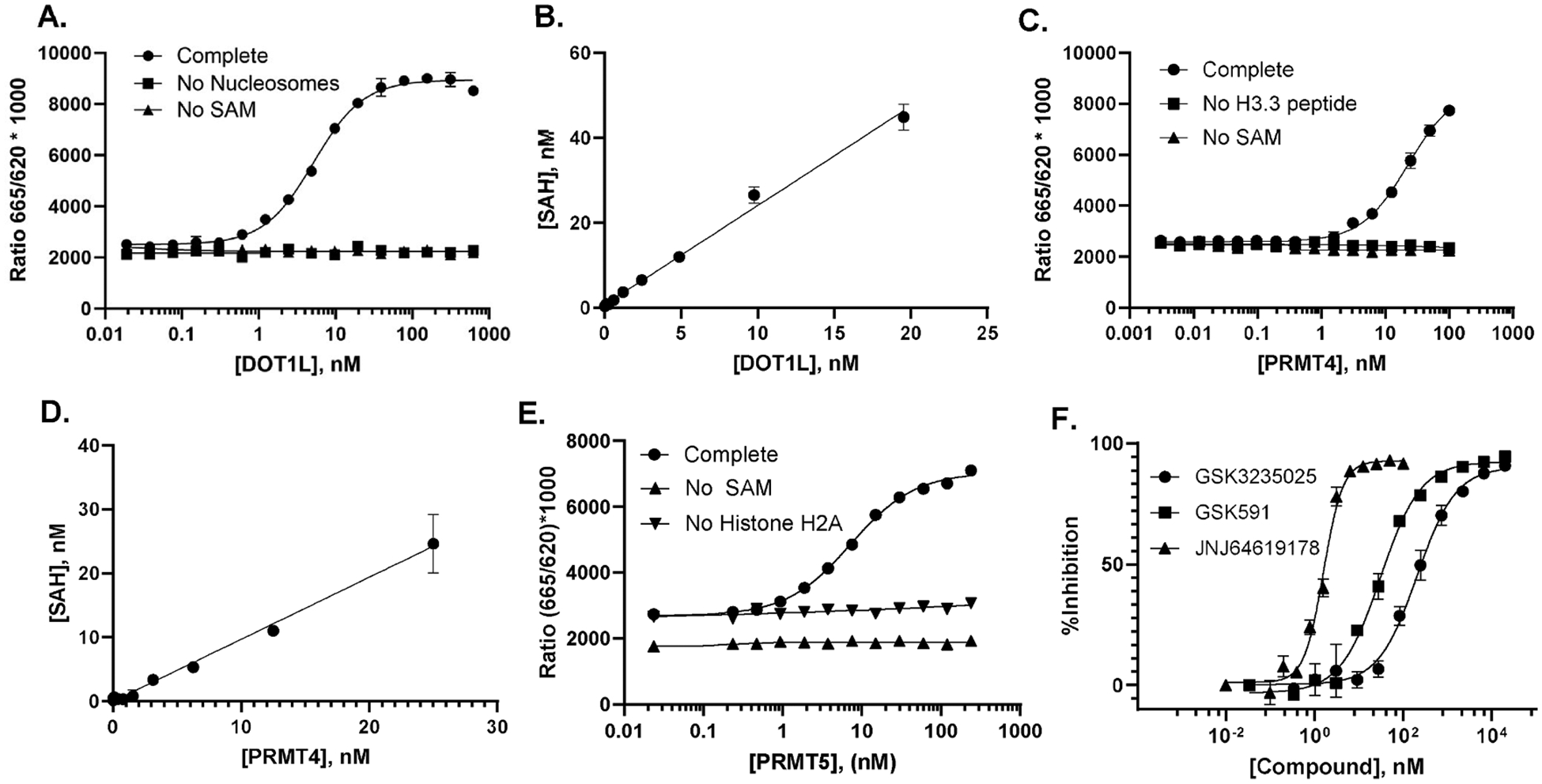
A. DOT1L enzyme titration. DOT1L was titrated in the presence of 110 nM SAM and 0.025 mg/mL Nucleosomes (Hela Mono/Di) 10 μM; reactions were incubated at 30 °C for 2 hr. B. SAH quantification. TR-FRET values were converted to SAH production using a standard curve. C. PRMT4 enzyme titration. PRMT4 was titrated in the presence of 245 nM SAM and 1 μM Histone H3 peptide; reactions were incubated at 30 °C for 120 min. D. SAH quantification, PRMT4. E. PRMT5 enzyme titration. PRMT5 was titrated in the presence of 5 μM SAM and 5 μM Histone H2A; reactions were incubated at 30 °C for 90 min. F. Dose response curves with probe inhibitors for PRMT5. Performed under initial velocity conditions as described for E, above. GSK3235025, GSK591, and JNJ64619178 showed IC_50_ values of 207 nM, 30.7 nM, and 1.59 nM, respectively.

For the three histone methyltransferases, we observed a robust signal in the AptaFluor assay that was strictly dependent on the presence of both SAM and methyl acceptor (Fig 4. A., C., E.). This becomes important with slow enzymes, where product formation may be substoichiometric with enzyme, e.g., PRMT 4 (Fig. 4.D.) and enzyme or substrate-bound SAH can contribute significantly to background signal. Conversion of the TR-FRET signal to SAH formation showed a linear response to enzyme up to at least 10 % SAM consumption, indicating initial velocity conditions (Fig. 4. B., D.). Despite the slow turnovers for these enzymes (Table 1), the EC_80_ concentrations, which yield robust signals under initial velocity conditions, were all less than 100 nM (Table 1), ensuring that the assay can be used to measure accurate IC_50_ values for inhibitors with potencies two to three times lower than that. For example, a dose response for the highly potent PRMT5 inhibitor JNJ64619178 yielded an IC_50_ of 1.59 nM (Fig. 4.F.), which compares favorably with the published ${\text{k}}_{\text{i}}^{\text{App}}$ of 0.772 nM determined by SAH detection using RapidFire Mass Spectrometry (MS) [13]. Similarly, our IC_50_ value of 30 nM for inhibition of PRMT5 by GSK591 agrees well with the published value of 22 nM [36].

**Fig. 5.**
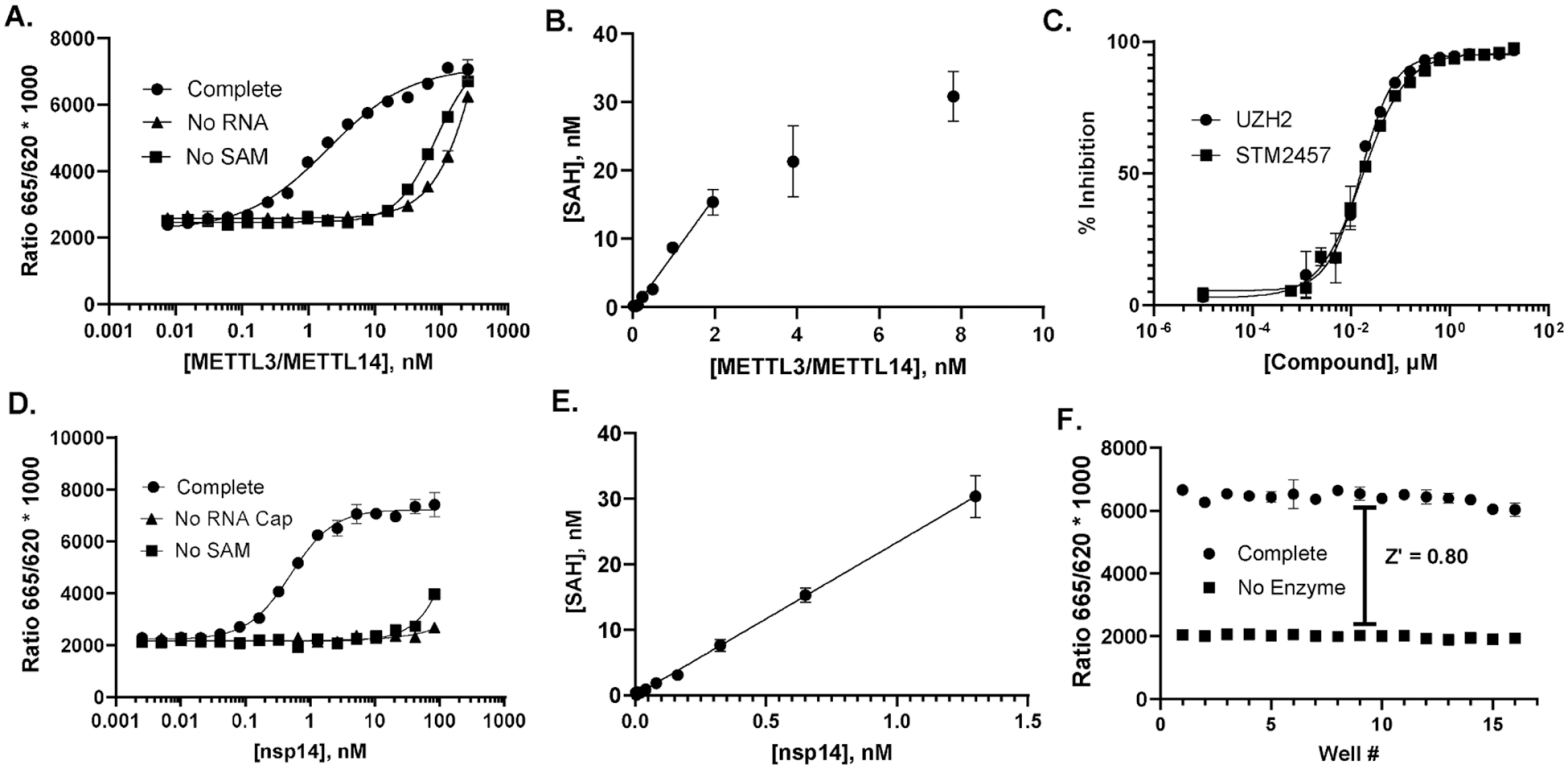
A. METTL3/METTL14 enzyme titration. METTL3/METTL14 was titrated in the presence of 250 nM SAM and 1 μM ssRNA 28mer; reactions were incubated at 30 °C for 2 hr. B. SAH quantification. TR-FRET values were converted to SAH production using a standard curve. C. Dose response curves with probe inhibitors for METTL3/METTL14. Performed under initial velocity conditions as described for A, above. UZH2 and STM2457 showed IC_50_ values of 16 nM and 17 nM, respectively. D. nsp14 enzyme titration. Nsp14 was titrated in the presence of 100 nM SAM and 50 μM RNA Cap; reactions were incubated at 37 °C for 90 min. E. SAH quantification. TR-FRET values were converted to SAH production using a standard curve. F. Z’ determination. Measured using initial velocity conditions: 1.45 nM nsp14, 90 min reaction.

The RNMTs we tested exemplify the combination of slow turnover and low SAM K_m_ exhibited by HMTs. METTL3/METTL14 has a reported SAM K_m_ of approximately 200 nM with an ssRNA substrate similar to the 28mer that we used [37]. At 250 nM SAM, we observed a robust signal with less than 10 nM METTL3/METTL14 in a two-hour reaction, with an optimal response (EC_80_) at 14 nM (Fig. 5A). There was a substantial background signal at higher METTL3/METTL14 concentrations, independent of either SAM or RNA substrate, possibly due to carryover of enzyme-bound SAH. Even so, there was a sufficient window for screening at the EC_80_ concentration of 14 nM. Conversion of the TR-FRET signal to SAH formation indicated a rate of 0.069 min^−1^ (Fig. 5B, Table 1) which is consistent with a reported kcat of 0.2 min^−1^ obtained using a radioassay with saturating SAM [37]. The reaction proceeded linearly up to 2 nM, well below the EC_80_, followed by a gradual decrease in rate, despite < 1 % depletion of SAM or methyl acceptor. The rate decrease could be a result of product inhibition, which is common for SAM-dependent MTs and has been reported for METTL3/MEETL14 [37,38]. Despite the non-linearity, the probe inhibitors UZH2 and STM2457 tested in dose response mode showed IC_50_ values of 16 nM and 17 nM, respectively (Fig. 5C), in reasonable agreement with published values of 5 and 17 nM, respectively [39,40].

**Fig. 6.**
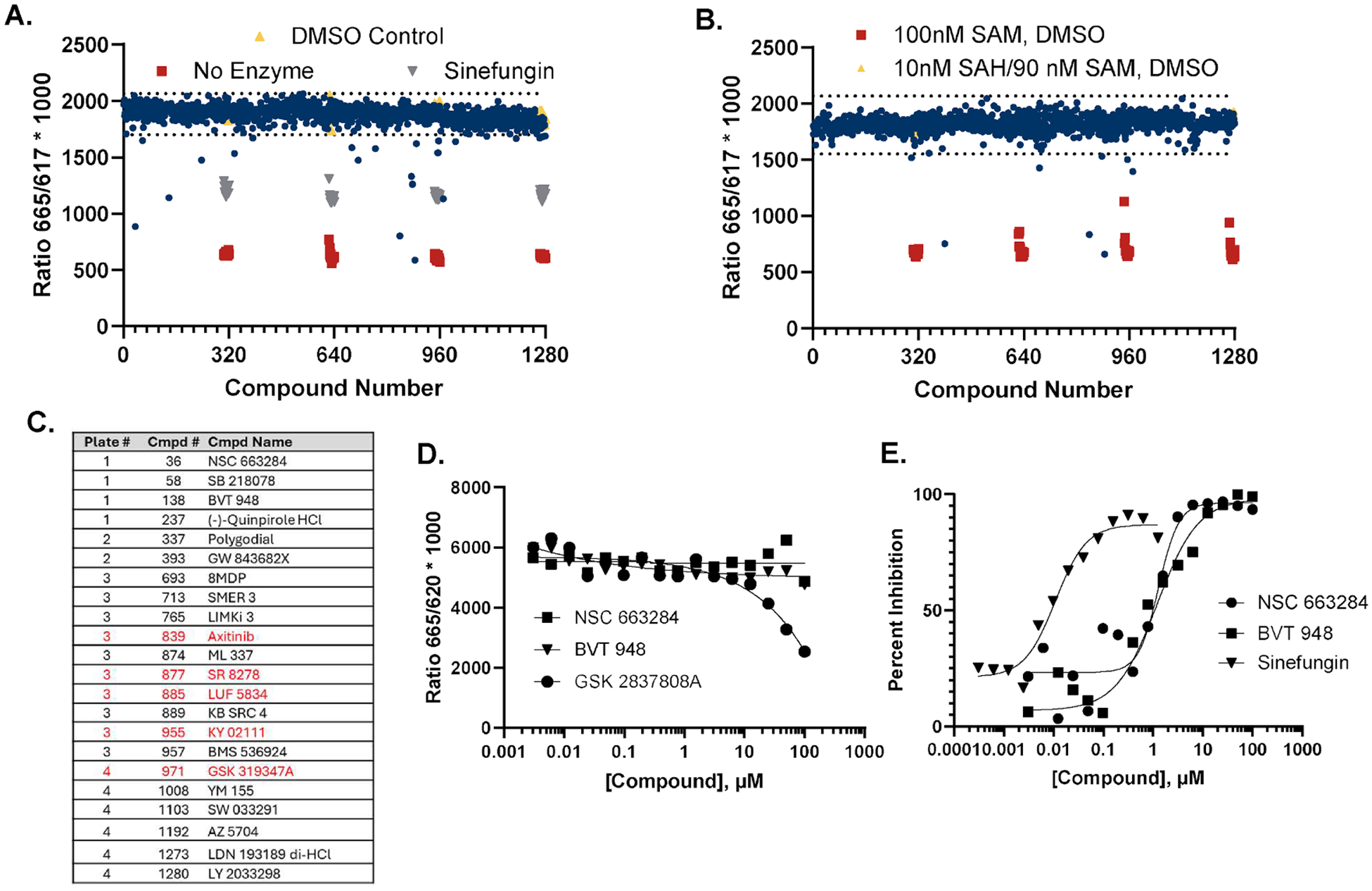
A. nsp14 pilot screen. 1280 bioactives were screened at 10 μM vs. nsp14 reactions under initial velocity conditions using the AptaFluor SAH assay. B. Interference screen. The 1280 bioactives were also screened vs. mock reactions mimicking completed nsp14 reactions (90 nM SAM, 10 nM SAH). C. List of nsp14 screening hits. Compounds that were also hits in the interference screen are in red type. D. Interference dose response assay. Two nsp14 hits were tested for interference with the AptaFluor assay in dose response. One of the hits from the interference screen (GSK2837808A) was used as a positive control. E. Dose response assay for two nsp14 screening hits and probe inhibitor, sinefungin.

Nsp14 has a reported SAM K_m_ of 257 nM [14]; we measured a K_m_ of 60 nM (data not shown) and used 100 nM for the ensuing assay development. We observed a robust response in the AptaFluor assay over a range of 0.2 to 5 nM nsp14, with an EC_80_ of 1.45 nM, and SAH formation was linear with enzyme well past 10 % depletion of SAM (Fig. 5D, E). The calculated rate of 0.26 min^−1^ at sub-saturating SAM is consistent with a reported k_cat_ of 0.87 min^−1^ determined using a radiometric assay [14]. In this case, there was a strong dependence on both SAM and the methyl acceptor, G(5′)ppp(5′)A, even at very high enzyme concentration (Fig 5D), and a Z’ of 0.80 indicated a high quality assay (Fig. 5F.). The SAM analog sinefungin was tested in dose response mode and showed an IC_50_ of 28 nM (Fig. 6E), in good agreement with a previously reported value of 20 nM [14].

To validate the AptaFluor SAH assay for HTS, we performed a pilot screen of 1280 bioactives with nsp14 at 1.45 nM, resulting in approximately 20 % conversion of the 100 nM SAM present. There was a very good signal window, with a tight clustering of most points around the DMSO controls, as reflected in a Z’ value of 0.86 (Fig. 6.A.). There were 22 hits, defined as compounds more than three standard deviations from the mean of the DMSO controls, for a hit rate of 1.7 % (Fig. 6.C.). In parallel, we ran an interference screen to identify false positives using mock reactions lacking nsp14 and containing 90 nM SAM and 10 nM SAH. Eight compounds appeared as hits in the mock reactions, five of which were also hits in the live screen (Fig. 6.B. and 6.C, red type). We tested two of the non-interfering hits, NSC-663284 and BVT-948, in dose response mode in mock reactions along with the false positive GSK-2837808A as a control (Fig. 6.D.). The non-interfering hits had no effect up to a concentration of 100 μM, whereas GSK-2837808A caused a dose dependent decrease in signal, confirming interference. In live reactions, both NSC-663284 and BVT-948 showed a dose-dependent response, confirming their inhibition of NSP-14 enzymatic activity (Fig. 6.E.).

## 4. Conclusion

The exquisite affinity and selectivity of a microbial SAH riboswitch enabled us to develop a generic assay for methyltransferases with the sensitivity to match their unusual kinetic constraints and the performance required for HTS and hit-to-lead work. In addition, by enabling direct, homogenous SAH detection rather than relying on coupling enzymes to convert SAH to a detectable analyte [17–24], the AptaFluor SAH assay eliminates a major source of compound interference, as reflected in the relatively low rate of false positives in an interference screen of bioactive compounds.

It is tempting to speculate on how microbial riboswitches might be used to develop HTS assays for other challenging enzymes that produce small molecule substrates. More than 50 naturally occurring riboswitches have been discovered that bind diverse metabolites, including nucleotides, amino acids, and even metal ions, e.g., flavin adenine mononucleotide (FMN), glycine, lithium [27]. However, they have evolved to recognize their ligands in the context of the cellular milieu, and their properties may not align with those needed for an enzymatic assay. For example, SAM aptamers have much lower affinity for their cognate ligand than SAH aptamers and are far less selective [41], probably because the intracellular SAM concentration is much higher than SAH. Recent efforts to develop riboswitch-inspired aptamers for new ligands using in vitro selection may overcome some of these limitations [42].

## Figures and Tables

**Table 1:** Summary of optimized AptaFluor MT reactions, including methyl acceptors, SAM concentrations (sub-saturating), optimal enzyme concentrations (EC_80_), and measured enzymatic rates.

Enzyme	Methyl Acceptor	[SAM] (nM)	EC_80_ (nM)	Rate (min^−1^)
**PRMT4**	Histone H3 (aa 1–21)	240	73.2	0.008
**PRMT5**	Histone H2A	5000	26.7	0.063
**DOT1L**	Nucleosomes	110	14.6	0.019
**METTL3/METTL14**	ssRNA (28mer)	250	14.3	0.069
**nsp14**	G(5′)ppp(5′)A	100	1.45	0.26

## Abbreviations:

DNMTs: DNA methyltransferases
EC_80_: enzyme concentration that produces 80% of maximum response
FP: fluorescence polarization
TR-FRET: Förster resonance energy transfer
HMTs: histone methyltransferases
MT: methyltransferase
RNMTs: RNA methyltransferase
SAH: *S*-adenosylhomocysteine
SAM: *S*-adenosylmethionine
